# Pseudodominant Nanophthalmos in a Roma Family Caused by a Novel *PRSS56* Variant

**DOI:** 10.1155/2020/6807809

**Published:** 2020-05-10

**Authors:** Lubica Dudakova, Pavlina Skalicka, Olga Ulmanová, Martin Hlozanek, Viktor Stranecky, Frantisek Malinka, Andrea L. Vincent, Petra Liskova

**Affiliations:** ^1^Research Unit for Rare Diseases, First Faculty of Medicine, Charles University and General University Hospital in Prague, Ke Karlovu 2, 128 08 Prague, Czech Republic; ^2^Department of Ophthalmology, First Faculty of Medicine, Charles University and General University Hospital in Prague, U Nemocnice 2, 128 08 Prague, Czech Republic; ^3^Department of Neurology and Centre of Clinical Neuroscience, First Faculty of Medicine, Charles University and General University Hospital in Prague, Katerinska 30, 120 00 Prague, Czech Republic; ^4^Department of Ophthalmology, Second Faculty of Medicine, Charles University and Motol University Hospital, V Uvalu 84, 150 06 Prague, Czech Republic; ^5^Ophthalmology Department, Third Faculty of Medicine, Charles University and Teaching Hospital Kralovske Vinohrady, Srobarova 1150/50, 100 34 Prague, Czech Republic; ^6^Department of Computer Science, Czech Technical University in Prague, Karlovo Namesti 13, 121 35 Prague, Czech Republic; ^7^Department of Ophthalmology, New Zealand National Eye Centre, University of Auckland, Private Bag 92019, Auckland 1142, New Zealand

## Abstract

**Background:**

The aim of the study was to identify the molecular genetic cause of two different Mendelian traits with ocular involvement present in the members of a single consanguineous Czech Roma family.

**Methods:**

We have performed ocular examination and review of medical records in two individuals diagnosed with nanophthalmos (proband and her father) and one individual followed for bilateral congenital cataract and microcornea (uncle of the proband). DNA of subjects with nanophthalmos was analysed by exome sequencing. Sanger sequencing was applied for targeted screening of potentially pathogenic variants and to follow segregation of identified variants within the family.

**Results:**

A homozygous variant c.1509G>C; p.(Met503Ile), in *PRSS56* was found in the two individuals affected with nanophthalmos. The change was absent from the gnomAD dataset, but two out of 118 control Roma individuals were also shown to be heterozygous carriers. Analysis of single nucleotide polymorphisms in linkage disequilibrium with the c.1509G>C in *PRSS56* suggested a shared chromosomal segment. The nanophthalmos phenotype, characterized in detail in the younger individual, encompassed bilateral corneal steepening, retinal folds, buried optic head drusen, and restricted visual fields, but no signs of retinal dystrophy. A known pathogenic founder *CTDP1* variant c.863+389C>T in a homozygous state was identified in the other family member confirming the suspected diagnosis of congenital cataracts, facial dysmorphism, and demyelinating neuropathy syndrome.

**Conclusions:**

Herein, we report the first occurrence of nanophthalmos in the Roma population. We have identified pseudodominant inheritance for this phenotype caused by a novel variant in *PRSS56*, representing a possible founder effect. Despite advances in genetic technologies such as exome sequencing, careful phenotype evaluation in patients from an isolated population, along with an awareness of population-specific founder effects, is necessary to ensure that accurate molecular diagnoses are made.

## 1. Introduction

The European Roma population originates from a small group of ancestors and displays a strong degree of endogamy, which has led to founder effects creating a unique genetic profile of subpopulation isolates [1].

Nanophthalmos represents a range of disorders with a small but structurally normal eye, as a result of ocular growth arrest. The clinical spectrum comprises global reduction in size or shortening of either the anterior or posterior segments of the eye, i.e., anterior and posterior microphthalmos. Nanophthalmos is a challenging condition for management as patients suffer from high hyperopia and a high incidence of angle-closure glaucoma, and occasionally pigmentary retinopathy is observed [2]. Prior classifications distinguished nanophthalmos from posterior microphthalmos based on normal corneal size and anterior chamber depth in the latter. However, as molecular genetic findings have demonstrated that both conditions can be caused by variants in the same genes, it has been suggested that they represent a spectrum of the same disease [3, 4].

To date, five genetic loci are reported to be linked to isolated nanophthalmos. Three types are inherited as an autosomal dominant trait; type 1 (NNO1; OMIM #600165) is associated with variants in the myelin regulatory factor (*MYRF*) gene [5]; type 3 (NNO3; OMIM %611897) has been mapped to chromosome 2q11-q14, and the disease-causing gene is yet to be discovered [6]; type 4 (NNO4; OMIM #615972) is caused by variants in the transmembrane protein 98 (*TMEM98*) gene [7]. Nanophthalmos type 2 (NNO2; #609549) and isolated microphthalmia type 6 (MCOP6; OMIM #613517) are autosomal recessive conditions associated with pathogenic variants in the membrane frizzled-related protein (*MFRP*) [8] and serine protease 56 (*PRSS56*) genes [9], respectively [10].

Congenital cataracts, facial dysmorphism, and neuropathy (CCFDN; OMIM #604168) is a unique syndrome, observed only in the Roma population, caused by a single nucleotide substitution in intron 6 of the C-terminal domain (CTD) phosphatase subunit 1 (*CTDP1*) gene [11, 12]. CCFDN is a rare multisystemic autosomal recessive disorder, which manifests with congenital cataracts variably associated with microphthalmia. Distinctive facial features become more apparent in early adulthood and include a prominent midface, a large nose, protruding teeth, and a small lower jaw. Progressive peripheral neuropathy in CCFDN starts early in life, leading to motor developmental delay. By adulthood, significant mobility difficulties are typically present [13]. Cognitive impairment is also invariably found in CCFDN patients [14]. CCFDN has been originally described in the Bulgarian Roma population; later reports indicate its presence in Roma individuals living in other countries, including the Czech Republic with 10 reported pediatric cases [11, 12, 15].

In this study, we performed molecular genetic investigation in a branch of a large consanguineous Roma family, with three individuals affected by two different monogenic traits with ocular involvement: nanophthalmos and CCFDN. We also provide a detailed description of the nanophthalmos phenotype.

## 2. Materials and Methods

The study was approved by the Ethics Committee of General University Hospital in Prague (reference no. 65/16) and adhered to the tenets set out in the Helsinki Declaration. All participants signed informed consent prior to the inclusion into the study.

Ocular examination included best-corrected visual acuity (BCVA) presented as decimal values. Corneal diameter, anterior chamber depth, and axial length were measured with the IOLMaster V.5 (Carl Zeiss Meditec AG, Jena, Germany), and corneal curvature and thickness were measured with the Pentacam (Oculus Optikgeräte GmbH; Wetzlar, Germany). The visual field was assessed with an automated perimeter (M-700; Medmont International, Vermont, Australia). High-resolution spectral-domain optical coherence tomography (SD-OCT) (Spectralis; Heidelberg Engineering GmbH, Heidelberg, Germany) was used to visualize macular architecture, fundus autofluorescence, and retinal nerve fibre layer (RNFL) thickness. B-scan ultrasound (Eye Cubed; Ellex, Adelaide, Australia) was undertaken to assess the optic head nerve or axial length. Standard neurological examination was performed in one subject.

Genomic DNA was extracted from peripheral lymphocytes in all available family members using the Gentra Puregene Blood Kit (Qiagen, Hilden, Germany).

Two family members with a clinical diagnosis of nanophthalmos (IV:4 and V:1) and one diagnosed with CCFDN (IV:1, Figure 1(a)) initially underwent genetic investigation. Whole exome sequencing (WES) was performed in those two with nanophthalmos phenotype. In individual V:1, DNA was captured using SureSelect^XT^ Human All Exon V6 (Agilent Technologies, Santa Clara, CA, USA) and sequenced on the HiSeq 2000 System (Illumina, Inc., San Diego, CA, USA). In individual IV:4, SeqCap EZ MedExome Target Enrichment Kit (Roche, Madison, WI, USA) was used and sequencing was performed on the Illumina NovaSeq 6000 Sequencing System.

Raw FASTQ reads were aligned to the human reference genome assembly GRCh37/hg19 using the Burrows–Wheeler Alignment tool [16]. Variant calling was performed with HaplotypeCaller [17]. A minimal coverage of 30x was achieved for >94.8% of the target sequence. Variants with a minor allele frequency (MAF) of less than 0.005 as per the gnomAD database [18], identified in 429 genes known to be associated with developmental and inherited eye disease, were filtered [19] and further evaluated for possible pathogenicity. The MAF threshold was chosen based on the extreme rarity of nanophthalmos. Six different software tools were applied to predict the pathogenicity of missense variants (Supplementary Table 2). Evolutionary amino acid conservation of the affected residue was visualized by multiple sequence alignment using T-Coffee [20]. Verification and segregation of the likely disease-causing variants within the family was performed by direct sequencing. Primer pairs used for PCR amplification and sequencing are listed in Supplementary Table 1.

Variant frequencies from 2,132 individuals of Caucasian Czech origin and 118 individuals of Roma descent living in the Czech Republic, gained through different next generation sequencing projects of the National Centre for Medical Genomics (NCMG, https://ncmg.cz/en), were used to assess population specific differences.

Classification of the detected genetic variants was performed based on the American College of Medical Genetics and Genomics (ACMG) guidelines [21] using an online tool [22].

**Figure 1:**
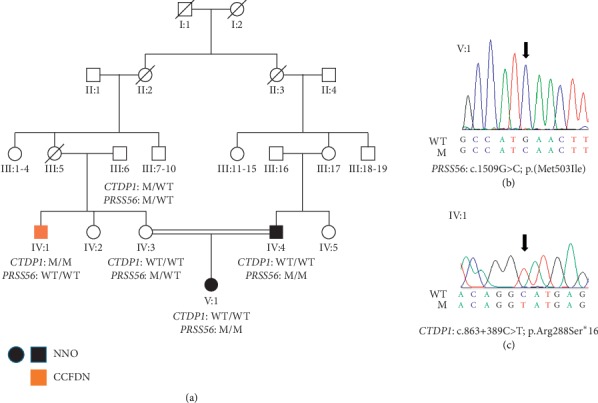
Segregation of the identified mutations. (a) Pedigree of the family, (b) sequence chromatogram of the homozygous variant c.1509G>C in *PRSS56* (NM_001195129.2), and (c) sequence chromatogram of the homozygous variant c.863+389C>T in *CTDP1* (NM_004715.4). CCFDN, congenital cataracts, facial dysmorphism, and neuropathy syndrome; M, mutation; NNO, nanophthalmos; WT, wild type.

To further explore whether the pathogenic variant underlying nanophthalmos is inherited from the same ancestor, we created, using WES data of individuals IV:4 and V:1 (Figure 1), a mini-haplotype with single nucleotide polymorphisms (SNPs) in linkage disequilibrium. Next, we established the possible presence of this mini-haplotype with a special focus on rare SNPs (minor allele frequency ≤0.09), considered to be highly specific markers, in WES data of two unrelated Roma control individuals, carrying the same heterozygous variant in *PRSS56*. Minimal coverage to consider the detected variants true was set to 15 as per visualization in Integrative Genomics Viewer (IGV) [23].

Based on a characteristic CCFDN phenotype in individual IV:1, we directly sequenced the noncoding region of the *CTDP1* (reference sequence NG_007988.1 and NM_004715.4), covering the known pathogenic variant c.863+389C>T, specific to the Roma population (Figure 1(a) and 1(c)).

## 3. Results

**Figure 2:**
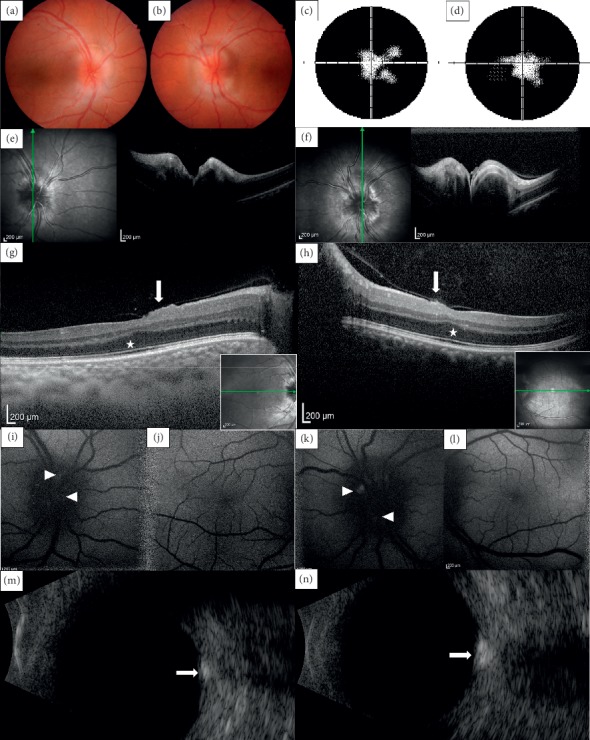
Posterior segment imaging in an individual with *PRSS56-*associated nanophthalmos. Fundus photograph of the right (a) and left (b) eye (age 11 years) abnormal optic discs with indistinct margins and no retinal pigmentary changes. Automated visual field testing within the central 50 degrees demonstrating irregular peripheral constriction in the right (c) and in the left (d) eye (age 16.5 years). Spectral domain optical coherence tomography scan of the right (e) and left (f) optic nerve heads (age 15 years), note abnormal elevation. Horizontal scans of the right (g) and left (h) macula, note absence of foveal depression and bilateral small papillomacular folds (arrows), no macular edema and thickened inner retinal and outer plexiform layers (asterisks). Autofluorescence imaging (age 16.5 years) of the right optic disc (i) and macula (j) and of the left optic disc (k) and macula (l); focal hyperfluorescence (arrowheads) suggests drusen, confirmed by B-scan ultrasonography in the right (m) and left (n) eye (arrows).

The proband, individual V:1 (Figure 1(a)), diagnosed with nanophthalmos, presented at the age of 2 years with right esotropia. Clinical notes documented bilateral horizontal papillomacular folds, optic head nerves with ill-defined borders, and tortuosity of retinal vessels at the age of 2 years and 10 months. Both eyes were highly hypermetropic (+10 diopter sphere (DS). Dilated fundus examination performed at the age of 11 years confirmed these findings, and no retinal pigmentary deposits were observed (Figures 2(a) and 2(b)). Sequential clinical findings are summarized in Table 1.

**Figure 3:**
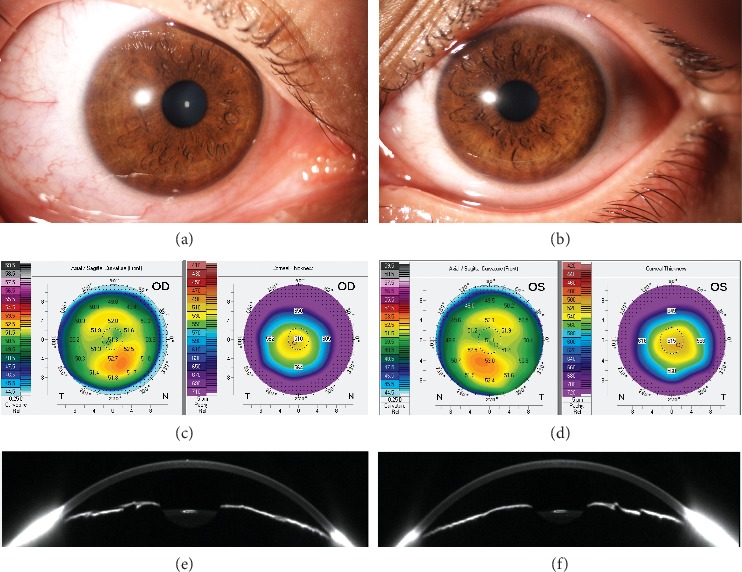
Anterior segment imaging in an individual with *PRSS56-*associated nanophthalmos. Slit-lamp photographs of the right (a) and left (b) eye appearing normal (age 16.5 years). Axial curvature and pachymetry maps of the right (c) and left (d) cornea (age 15 years) documenting high steepening and normal corneal thickness. Scheimpflug images of the horizontal cross sections of the right (e) and left (f) anterior segment showing narrow iridocorneal angles.

At the last examination, age 16.5 years, the patient denied nyctalopia. Her BCVA was 0.32 in the right eye and 0.4 in the left eye (Figures 3(e) and 3(f)). The anterior segment showed narrow iridocorneal angles. Both corneas had normal diameter and thickness, but were abnormally steep (Table 1; Figure 3(a) and 3(b)). Axial lengths were below 17 mm, confirming markedly shortened posterior segments in both eyes (Table 1). Constricted visual fields bilaterally, previously documented by kinetic perimetry at 12 years, were confirmed (Figures 2(c) and 2(d)). SD-OCT showed bilateral papillomacular folds with preserved stratification and attenuated foveal depression (Figures 2(e)–2(h)), a typical finding in eyes with nanophthalmos (8). RNFL was bilaterally increased (Figures 2(e) and 2(f)). Fundus autofluorescence showed bilateral optic head nerve drusen (Figures 2(i) and 2(k)) confirmed by B-scan ultrasound (Figures 2(m) and 2(n)). Dilated fundus examination, allowing for examination of the far periphery, was not performed at this point because of the risk of angle closure. The patient was otherwise developmentally normal, and electroencephalography performed at the age of 11 years was unremarkable.

Individual IV:4, father of V:1 (Figure 1(a)), also had nanophthalmos. Clinical note review documented that his BCVA was 0.66 in both eyes and intraocular pressure (IOP) bilaterally below 21 mmHg at age 29 years. Regular follow-up commenced at the age of 32 years because of angle-closure glaucoma in both eyes. IOP remained elevated in both eyes, despite intensive topical glaucoma medications, systemic dorzolamide, and extensive surgical interventions in the right eye (lens extraction, pars plana vitrectomy, and trabeculectomy with Ex-PRESS shunt).

Upon referral to a tertiary care hospital at the age of 35 years, BCVA was light projection in the right eye and 0.4 in the left eye, with +12 DS refractive error. The central corneal thickness measured was 526 *µ*m and 534 *µ*m in the right and left eye, respectively. IOP was 36 mmHg in the right eye and 26 mmHg in the left eye. Temporal angle closure was noted in the right eye. In the left eye, concentric constriction of the visual field was documented. The patient continued using three to four different topical glaucoma medications in addition to systemic dorzolamide. Pars plana lensectomy and vitrectomy with silicone oil was performed elsewhere, which was complicated by retinal detachment on the first postsurgical day. IOP remained elevated, and four laser iridotomies were applied; however, vision in the left eye could not be preserved, and by the age of 36 years, only light projection remained in the left eye, while the right eye was totally blind by this age. At the last follow-up examination, age 40 years, vision was no light projection bilaterally.

A novel homozygous variant, c.1509G>C; p.(Met503Ile), was detected in the *PRSS56* gene (reference sequence NM_001195129.2) by WES in individual V:1 (Figure 1(b)). The variant was evaluated as pathogenic or likely pathogenic by four out of the five in silico algorithms used (Supplementary Table 2) and fully conserved across 14 different species (Supplementary Figure 1). Targeted Sanger sequencing demonstrated that the father (IV:4), who was also affected with nanophthalmos, had the same *PRSS56* variant in a homozygous state, while her unaffected mother was a heterozygous carrier (Figure 1(a) and 1(b)). The c.1509G>C in *PRSS56* was absent from the gnomAD dataset, as well as in WES data from 2,132 individuals of Czech Caucasian origin. A different missense change affecting the same amino acid residue has been observed previously in a patient with nanophthalmos [27]. Given all this genetic evidence, the variant was interpreted as pathogenic according to the ACMG guidelines [21, 22].

No possibly pathogenic changes were found in coding sequences of other genes associated with nanophthalmos/microphthalmos. Other rare variants detected in the ocular gene panel in individual V:1 are listed in Supplementary Table 3.

**Figure 4:**
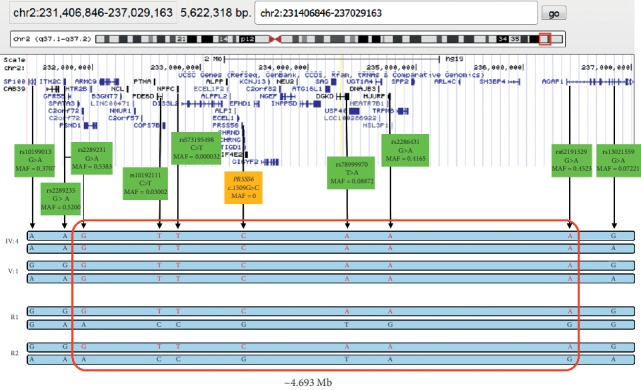
Putative ancestral chromosomal segment around the *PRSS56* c.1509G>C variant. The single nucleotide polymorphism (SNP) database reference SNP (rs) ID of each variant and its minor allele frequency (MAF) mined from gnomAD database v2.1.1. are shown. SNPs in linkage disequilibrium with the disease-causing variant in *PRSS56* as observed in two affected individuals (IV:4 and V:1) are highlighted in red. These SNPs were also found in two other reportedly unrelated Czech Roma subjects (R1 and R2), heterozygous carriers of the *PRSS56* variant, indicating putative shared chromosomal segment with minimal interval delineated by two low frequent SNPs, rs10192111 and rs78999970 (size ∼1.65 Mb). The region possibly extends up to variants rs2289231 and rs62191529 (size ∼4.69 Mb).

The c.1509G>C variant in *PRSS56* was, however, identified in two out of 118 Roma control subjects collected in the Czech Republic. A likely shared chromosomal segment extending possibly up to 4.69 Mb was identified by genotyping of SNPs in linkage disequilibrium (Figure 4). For this purpose, WES was also performed in individual IV:4 (affected father of the proband). In addition, analysis of his WES data did not lead to the identification of any other possibly pathogenic variants in genes implicated in nanophthalmos/microphthalmos, thus further supporting the pathogenicity of the detected *PRSS56* mutation.

Individual IV:1 (Figure 1(a)), uncle of the proband, had a history of bilateral congenital cataracts, microcornea, and horizontal nystagmus. Because of delayed psychomotor development, facial dysmorphism, intellectual disability (IQ 35–49), and severe peripheral polyneuropathy, the diagnosis of CCFDN has been suspected. A detailed clinical course and findings in this subject are provided in Supplementary Materials (available here). Despite being a relatively severe and well-defined syndromic disease, a definitive clinical diagnosis had not been made until our investigation. A known homozygous variant c.863+389C>T in the *CTDP1* gene was identified by targeted Sanger sequencing, confirming the diagnosis of CCFDN (Figure 1(c)).

The extended family was also reported to have a history of congenital deafness in two individuals and severe visual and neurological impairment variably associated with mental retardation in two other members, most likely additional cases with CCFDN.

## 4. Discussion

Herein, we expand the spectrum of *PRSS56* variants causing autosomal recessive nanophthalmos and describe the associated phenotype in two individuals from a consanguineous Czech Roma family.

Roma is a genetically isolated ethnic group derived from a limited number of founders, with the majority (about 8 million individuals) living in Europe [15, 28]. A number of recessive Mendelian disorders caused by private mutations have been described in this population [28].

As the c.1509G>C variant in *PRSS56* was found to be in linkage disequilibrium with several SNPs, including a change with very low frequency (MAF = 0.00032), in the two affected individuals and in two Roma control individuals, we hypothesize that they share an ancestral haplotype. Based on the carrier frequency in the controls (2 out of 236 alleles), obtained as anonymized data from NCMG, the estimated prevalence of nanophthalmos should be around 1 in 15,000 within the Czech Roma population. However, as the disease has not been, to the best of our knowledge, reported in Roma individuals, either the condition is mis- or underdiagnosed, and/or there is a deviation from Hardy–Weinberg equilibrium [29]. All of these factors would not be surprising due to the genetic and social isolation and poor socioeconomic status [30]. It should also be noted that our control dataset was of limited size, lacking details on geographical origin, which could have introduced bias, i.e., it cannot be excluded that the two heterozygous individuals carrying the c.1509G>C in *PRSS56* are members of the extended family. Thus, it remains to be elucidated if the *PRSS56* variant is private only to the particular Roma family or to the subisolate living in the Czech Republic, or if it can be found in other Roma subpopulations.

The mechanism by which sequence changes in *PRSS56* cause nanophthalmos has not been fully explained; it has been suggested the encoded protein is part of a complex regulatory network influencing postnatal eye development [31].

Except for corneal steepening, the anterior segment in individual V:1 appeared normal, such that in the past, she would be diagnosed with posterior microphthalmos [9]. RNFL measurements and visualization of optic discs corroborated previous observations that retinal folds in nanophthalmos spare the outer retina, except for the outer plexiform layer [32]. Corneal steepening has also been previously described in nanophthalmos, albeit not genetically refined [24].

Nanophthalmos is associated with a high rate of secondary angle-closure glaucoma and surgical complications [33]. Retrospective review of the disease course in individual IV:4 confirms these difficulties in clinical management.

Another family member, individual IV:1, had typical signs of the CCFDN syndrome which was confirmed by the identification of the known intronic founder mutation c.863+389C>T in *CTDP1,* leading to an activation of a cryptic splice acceptor site [12]. *CTDP1* encodes an enzyme phosphorylating serine residues in the CTD domain of RNA polymerase II subunit, the actual mechanism of CCFDN phenotype development remains however unknown [34]. The significant delay in a definitive diagnosis, not made until the age of 41 years, highlights the lack of awareness of professionals regarding disorders unique to patients of Roma descent, most likely accounting for prevalence underestimates [11, 15].

In summary, to the best of our knowledge, we report the first occurrence of nanophthalmos in the Roma population, caused by a novel variant in *PRSS56* representing a possible founder effect. Our study also demonstrates the importance of accurate phenotyping linked to population-specific knowledge.

## 5. Conclusion

Continuing characterization of Mendelian traits in population isolates is important for targeted variant detection, leading to better genetic counseling and increased rate of correctly diagnosed cases.

## Figures and Tables

**Table 1:** Ophthalmic examination results of individual V:1 with nanophthalmos caused by a homozygous variant in *PRSS56*.

Age (y)	BCVA		Refraction^†^ (DS/DC)		IOP (mmHg)		CCT (*µ*m)		WTW (mm)		K1/K2 (D)		AL (mm)		ACD (mm)	
	RE	LE	RE	LE	RE	LE	RE	LE	RE	LE	RE	LE	RE	LE	RE	LE
11	0.3	0.6	+12.5/+1.0 × 125°	+12.5	17	16	515	513	UA	UA	UA	UA	14.8	14.4	UA	UA
12	0.32	0.63	+13.75/−0.5°	+13.75/−0.5°	21	20	UA	UA	UA	UA	UA	UA	UA	UA	UA	UA
13	0.2	0.4	+13.75/−0.5°	+13.75/−0.5°	20	19	UA	UA	UA	UA	UA	UA	UA	UA	UA	UA
15	0.32	0.4	+13.75/−0.5°	+13.75/−0.5°	21	15	510	515	11.4	11.4	51.3/52.3	51.2/52.5	15.69	16.27	2.93	2.85
16.5	0.32	0.4	+13.5/−1.0	+13.5	19	14	UA	UA	11.3	11.5	UA	UA	16.36	16.26	2.72	2.89

ACD, anterior chamber depth (normal values 3.14 ± 0.33 mm) [24]; AL, axial length (normal values 23.42 ± 0.46 mm) [25]; BCVA, best-corrected visual acuity; CCT, central corneal thickness (normal values 552.6 ± 36.8 μm) [25]; D, diopter; DC, diopter cylinder; DS, diopter sphere; IOP, intraocular pressure; *K*1/*K*2, flat/steep keratometry readings (normal values *K*1 ≤46.1 D, *K*2 ≤47.4 D) [26]; LE, left eye; RE, right eye; y, years; UA, unavailable data; and WTW, white-to-white corneal diameter. ^†^subjective values.

## Data Availability

The clinical and genetic data used to support the findings of this study are included within the article and within the supplementary information files.

## Supplementary Materials

Supplementary Materials Supplementary Table 1: primer sequences and conditions used in this study. Supplementary Table 2: In silico analysis of the p.(Met503Ile) mutation in *PRSS56*. Supplementary Figure 1: Evolutionary amino acid conservation of affected residue across 14 species (T-Coffee alignments). Supplementary Table 3: Rare variants identified in genes associated with disorders affecting ocular development in individual V:1.
