# Supplementary material for: Pseudodominant Nanophthalmos in a Roma Family Caused by a Novel PRSS56 Variant
Source: J Ophthalmol. 2020 May 10;2020:6807809. doi: 10.1155/2020/6807809 (PMC7212339; doi:10.1155/2020/6807809)
Supplement: Supplementary Materials — Supplementary Table 1: primer sequences and conditions used in this study. Supplementary Table 2: In silico analysis of the p.(Met503Ile) mutation in PRSS56. Supplementary Figure 1: Evolutionary amino acid conservation of affected residue across 14 species (T-Coffee alignments). Supplementary Table 3: Rare variants identified in genes associated with disorders affecting ocular development in individual V:1. [file 6807809.f1.docx]

# SUPPLEMENTARY MATERIAL

# Detailed personal history and phenotype of individual IV:1 diagnosed with congenital cataracts, facial dysmorphism, and neuropathy syndrome.

# The patient underwent bilateral cataract surgery in infancy without artificial lens implantation. When he was 24 years old, an anterior chamber lens was introduced into the right eye. As a sequela he developed secondary glaucoma in the right eye for which he underwent trabeculectomy in the third decade of his life.

# At the age of 36 years he was referred to the Department of Ophthalmology, First Faculty of Medicine, Charles University and General University Hospital in Prague because of right corneal decompensation. When first examined, microcornea (corneal diameter 9.75 mm in both eyes) and horizontal nystagmus was documented, with visual acuity counting fingers in the right eye and light perception in the left eye. The right cornea was opaque due to edema, and in the left eye, ring synechia of the iris was present. Examination of the fundus was not possible due to the anterior segment pathology. Penetrating keratoplasty (PK) was performed in the right eye. The postoperative period was however complicated by graft rejection 11 months after PK, and elevated IOP managed by cyclophotocoagulation. At the last ocular examination, when the patient was aged 40 years, visual acuity was bilaterally hand movements. Ultrasound examination excluded microphthalmos (axial length 26.36 mm and 25.02 mm in the right and left eye, respectively) and retinal detachment in both eyes.

# The proband was known to have delayed psychomotor development. He also had a history of cryptorchism surgery in childhood. Facial dysmorphism, intellectual disability (IQ 35–49), obesity (body mass index 40 at the age of 36 years), difficulties with walking, back pain, hypertension and hearing impairment were noted during ophthalmic examinations.

# Clinical neurological examination performed at the age of 41 years confirmed severe peripheral polyneuropathy, with predominantly motor involvement, distal muscle weakness and in lower and upper limbs. Extensor plantar response, mild ataxia, and postural and kinetic tremor of the upper limbs were also present.

# Supplementary Table 1: Primers sequences and conditions used in this study.

|  | Forward | Reverse | Product size (bp) | Annealing temperature |
| --- | --- | --- | --- | --- |
| *CTDP1* c.863+389C>T | TCCTGACCTCGTGATCTTCC | CCTGGGAGACAGTGGGAGTA | 195 | 60°C |
| *PRSS56*  c.1509G>C | ACCGCAACCGTTCATTATTC | CCTCGGACCCTCTACCTACC | 301 | 60°C |

Reference sequences: NG_007988.1 (*CTDP1*); NM_001195129.1 (*PRSS56*)

# Supplementary Table 2: *In silico* analysis of p.(Met503Ile) mutation in *PRSS56*.

|  | **PolyPhen2** | **SIFT** | **MutPred2** | **SNP&GO** | **PhD-SNP** | **CADD** |
| --- | --- | --- | --- | --- | --- | --- |
| *PRSS56*  c.1509G>C | 0.883 | NA | 0.272 | NA | NA | 23.5 |
|  | Possibly damaging | Damaging | Neutral | Disease | Disease | Disease causing |

Reference sequence: NM_001195129.1 and NP_001356777.1, NA – not available

Overall probability MutPred score >0.5 was considered as probably disease-causing and a score > 0.75 was considered as disease-causing. For CADD score >20 was considered as pathogenic.

**Supplementary Figure 1: Evolutionary amino acid conservation of affected residue across 14 species (T-coffee alignments).**


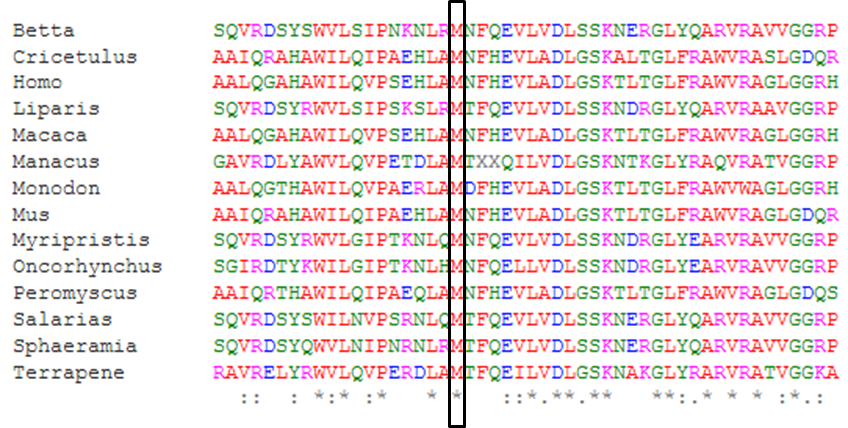


**Supplementary Table 3: Rare variants identified in genes associated with disorders affecting ocular development in individual V:1.**

| **Chromo-some** | **Position** | **Zygosity** | **Gene and**  **RefSeq ID** | **Variant description** | **rs ID** | **gnomAD frequency**  **# of alleles het/hom/total** | **Associated phenotype**  **OMIM number** | **Inheritance** |
| --- | --- | --- | --- | --- | --- | --- | --- | --- |
| 1 | 150525555 | het | *ADAMTSL4*  NM_001288607.2 | c.260G>A; p.(Arg87Gln) | rs199599791 | 0.000168  47/ 0 / 278,742 | Lens ectopia  #225200 | AR |
| 2 | 36583511 | het | *CRIM1*  NM_016441.3 | c.76C>A; p.(Leu26Met) | rs534996132 | 0.003545  861 / 30 / 242,846 | Macrophthalmia, coloboma, microcornea  #602499 | AD |
| **2** | **233389542** | **hom** | ***PRSS56***  **NM_001195129.1** | **c.1509G>C; p.(Met503Ile)** | **0** | **0** | **Microphthalmia**  **#613517** | **AR** |
| 3 | 49167182 | hom | *LAMB2*  NM_002292.3 | c.1406-33T>C; p.? | rs557786607 | 0.000874  246 / 7 / 281,602 | Pierson syndrome  #609049 | AR |
| 4 | 8869701 | het | *HMX1*  NM_018942.3 | c.765C>T; p.(Arg255Arg) | rs574706311 | 0.000540  101 / 0 / 186,972 | Oculoauricular syndrome  #612109 | AR |
| 4 | 183676354 | het | *TENM3*  NM_001080477 | c.4834G>A; p.(Gly1612Ser) | rs543733302 | 0.000297  73 / 2 / 245,596 | Microphthalmia with coloboma  #615145 | AR |
| 9 | 100227317 | het | *TDRD7*  NM_014290.2 | c.1629+7A>G; p.? | rs151288166 | 0.002321  649 / 4 / 279,676 | Cataract  #613887 | AR |
| 14 | 70490008 | het | *SMOC1*  NM_001034852.2 | c.1135A>C; p.(Ile379Leu) | rs766710070 | 0.000056  14 / 0 / 251,432 | Microphthalmia with limb anomalies  #206920 | AR |
| 14 | 74971592 | het | *LTBP2*  NM_000428.2 | c.4370-28C>T; p.? | rs143626054 | 0.000880  249 / 1 / 282,762 | Microspherophakia/megalocornea,  with ectopia lentis, with or without secondary glaucoma  #251750 | AR |
| 14 | 75019032 | het |  | c.1257C>T; p.(Pro419Pro) | rs150977144 | 0.000036  9 / 0 / 247,760 |  |  |
| 14 | 75052587 | het |  | c.800C>T; p.(Ser267Leu) | rs149952751 | 0.000975  274 / 1 / 280,846 |  |  |
| 16 | 77353778 | het | *ADAMTS18*  NM_001326358.2 | c.1984C>T; p.(Pro662Ser) | rs139553755 | 0.001966  555 / 5 / 282,356 | Microcornea, myopic chorioretinal atrophy and telecantus  #615458 | AR |
| 17 | 79479366 | het | *ACTG1*  NM_001199954.2 | c.15C>T; p.(Ile5Ile) | rs199657153 | 0.001287  364 / 7 / 282,748 | Baraitser/Winter syndrome 2  #614583 | AD |
| 20 | 17475569 | het | *BFSP1*  NM_001161705.2 | c.773A>G; p.(Asn258Ser) | rs150798461 | 0.002419  684 / 16 / 282,738 | Cataract  #611391 | AD, AR |

het = heterozygous, hom = homozygous, AR = autosomal recessive, AD = autosomal dominant, rs ID = dbSNP reference SNP number , OMIM = Online Mendelian Inheritance in Man

**Web resources:**

PolyPhen2 (Polymorphism Phenotyping v2): http://genetics.bwh.harvard.edu/pph2/

SIFT (Sorting Intolerant From Tolerant): http://sift.jcvi.org/

MutPred2: http://mutpred.mutdb.org/

SNPs&GO; PhD-SNP: http://snps.biofold.org/snps-and-go/

CADD https://cadd.gs.washington.edu/snv

T-coffee: http://tcoffee.crg.cat/apps/tcoffee/index.html

gnomAD: https://gnomad.broadinstitute.org/

OMIM: https://www.omim.org/

dbSNP: https://www.ncbi.nlm.nih.gov/snp/
